# Dislocation Multiplications in Extremely Small Hexagonal-structured Titanium Nanopillars Without Dislocation Starvation

**DOI:** 10.1038/s41598-017-16195-7

**Published:** 2017-11-21

**Authors:** Peng Huang, Qian Yu

**Affiliations:** Center of Electron Microscopy and State Key Laboratory of Silicon Materials, Department of Materials Science and Engineering, Zhejiang University, Hangzhou, 310027 China

## Abstract

“Smaller is stronger” has been commonly observed in cubic structured and hexagonal close-packed (HCP) structured materials. Dislocation starvation phenomenon is highly responsible for the increase of strength at smaller scale in cubic materials. However, by using quantitative *in situ* transmission electron microscope (TEM) nano-mechanical testing on cylindrical titanium nano-pillars with diameters of ~150 nm but varied orientations and three dimensional dislocation tomography, we found that dislocation nucleation and multiplication dominate the plastic deformation of the nano-pillars with no sign of dislocation starvation, resulting in much better ability of dislocation storage and plastic stability of HCP structured materials at extremely small scale.

## Introduction

Mechanical properties and plastic deformation of metals at small scale have received considerable attention as the development of MEMS and micron/nano mechanics^1–6^. It has been found that there is a strong size effect which shows a reproducible trend that “smaller is stronger” as the diameter of the samples reduce from conventional sizes (millimeters and above) into micrometer and submicron regime in single crystal body-centered cubic (BCC) and face-centered cubic (FCC) metals, as well as hexagonal close-packed (HCP) metals^7–14^. For small-volume BCC and FCC metals, dislocation starvation and source exhaustion are the main mechanisms used to explain the size effect^15–18^. When the sample size is very small, mobile dislocations escape from the crystal at the nearby free surfaces before multiplying and interacting with other dislocations due to the presence of image stress field; higher stress is required for the activation of secondary easy slip systems or nucleation of dislocations. The easy glide of dislocations in small volumes makes dislocation storage difficult and usually facilitates strain bursts. Since both the driving force—the image stress and the critical stress needed for dislocation self-multiplication increase with decreasing size, as the external size is reduced to a critical value (~200 nm for FCC and BCC samples) the specimen eventually loses the ability for dislocation storage and the entire structure catastrophically collapses under loading.

In contrast, the dislocation starvation phenomenon was rarely reported in HCP structured materials. Titanium, magnesium and their alloys with different sizes and different orientations have been studied. For example, Sun *et al*.^10^ tested the size effect on double-slip mechanism in titanium. While the size effect in strength was observed, deformation mode of double slip was effective even as the sample size reduced to 350 nm. The [0001] oriented titanium and magnesium single crystals were also studied in which the activation of dislocation slip would be more difficult since the shear stress barely resolved onto the easy glide systems. Under this extreme condition, the uncommon 〈c + a〉 dislocations’ activities were observed in ~200 nm sized samples as well^19^. However, the detailed and dynamic behaviors of 〈a〉 dislocations (major type of mobile dislocations in HCP materials) at small scale, remain unclear. It would be then interesting to study if dislocation starvation phenomenon exists in HCP metals and the intrinsic dislocation behaviors in extremely small volume, which would not only help our understanding on the intrinsic dislocation behaviors at small scale but also shed light on future application of small crystals in MEMS.

By using quantitative *in situ* transmission electron microscope (TEM) nano-mechanical testing technique and three-dimensional dislocation tomography, we characterized the deformation of extremely small Ti-5at%Al single crystals with different orientations to systematically investigate the dislocation behaviors at small scale and the plastic stability of this HCP structured material. The nano-pillars with diameter of ~150 nm were fabricated by focused ion beam (FIB) and loaded 5 degrees off the [$10\bar{1}0$] direction and 10 degrees off the [0001] direction, respectively. By using a Hysitron Nano indentation outfitted with a flat punch diamond tip, compression tests were performed *in situ* inside a JEOL 3010 TEM.

High density of dislocations were observed with the formation of complex dislocation network in the nano-pillars compressed 5 degrees off [$10\bar{1}0$]. The pillars exhibit high strength (5 GPa) and continuous plastic flow, dislocations that formed the dislocation net were all 〈 a 〉 types with Burgers vector $\pm {\rm{a}}/3$ [$2\bar{1}\bar{1}0$], $\pm {\rm{a}}/3$[$\bar{1}\bar{1}20$] and $\pm {\rm{a}}/3$[$\bar{1}2\bar{1}0$], originally nucleated from the contact surface. As the pillar was compressed 10 degrees off the [$0001$] direction, a dominant dislocation source was activated that continuously generated the same type of dislocations, forming a dislocation array. The pillar showed similar level of strength and continues plastic flow without significant dislocation bursts. In either case, dislocation starvation was not observed, which was completely different from FCC and BCC metals.

## Results and Discussion

**Figure 1:**
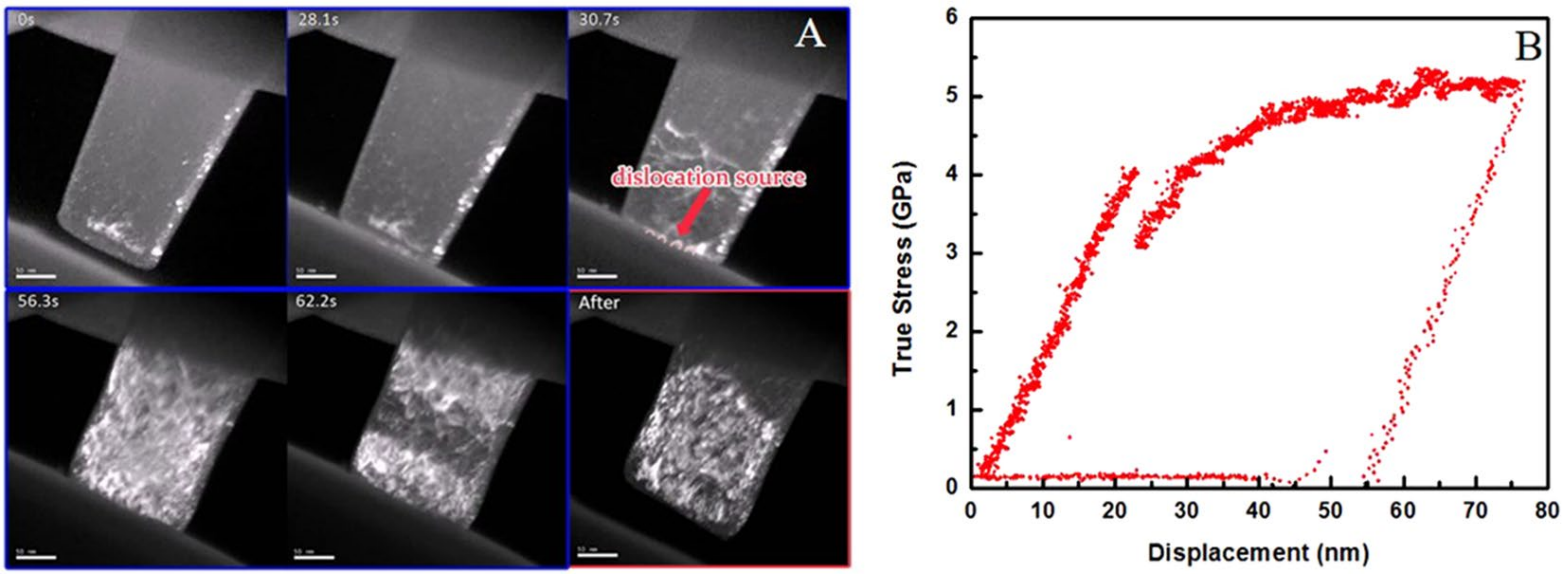
(**A**) Sequential TEM images from the movie of the *in situ* compression test, the loading direction was 5 degrees off [$10\bar{1}0$], (**B**) True stress-displacement curve of the quantitative *in situ* TEM nano-mechanical test. Figure 1B shows the continuous plastic flow and high strength after deformation.

Figure 1A shows a dark field TEM image of a Ti alloy nano-pillar with a diameter of ~150 nm, the compression axis of which was 5 degrees off [$10\bar{1}0$]. Its corresponding stress-displacement curve is plot in Fig. 1B. As shown in the *in situ* compression movie (supplementary movie 1), no obvious dislocation contrast was observed in the nano-pillar at the beginning; with further loading, several dislocation sources (marked by red dash lines) were identified near the contact surface which suddenly started to emit dislocations as the contact stress raised to ~4 GPa. The nucleation of multiple dislocations was also followed by a stress drop. In Fig. 1B, a ~1 GPa load drop was observed accordingly. The dislocations quickly intersected with each other and formed junctions. As the applied force increased, more dislocations were formed from the dislocation network, resulting in a very high density of dislocations in the extremely small nano-pillar. Subsequently, the mechanical curve displays a strain-hardening with continuous plastic flow; no significant stress serration was observed as that commonly happens in FCC and BCC nano-pillars. Consistent with the mechanical data, the plastic deformation of the Ti nano-pillar was also continuous. No obvious large shear steps were observed from the surface and the entire deformation was homogeneous. It is indicated that the extremely small-sized HCP Ti nano-pillars are able to store dislocations and sustain dislocation interactions and multiplications. Better plastic stability is achieved compared to FCC and BCC small crystals.

**Figure 2:**
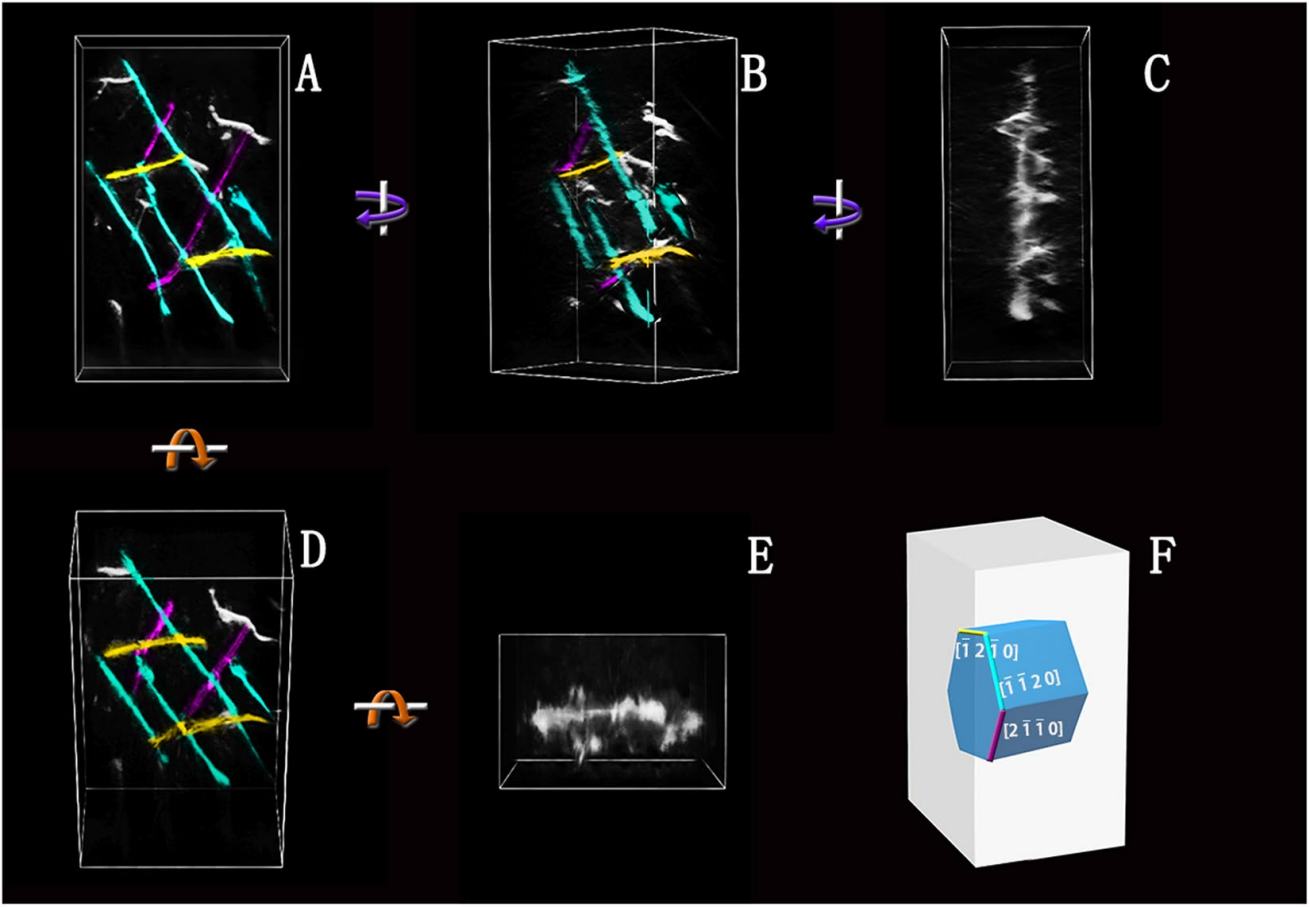
(**A**)–(**E**) Views of the 3D-dislocation net model, Showing that the dislocations were all <a> types with Burgers vectors $\pm a/3\,[2\overline{1}\overline{1}0],\pm a/3\,[\overline{1}\overline{1}20]\,{\rm{and}}\pm a/3\,[\overline{1}2\overline{1}0]$, respectively. (**F**) Schematic diagram that illustrates the loading condition.

The dislocation network formed right after yielding was the critical structure that facilitated the further dislocation interaction and multiplications. To characterize the dislocation network structure, dislocation tomography technique was applied to generate the real three dimensional structure. The tested nano-pillar had the similar size and the same orientation as the pillars above. It was compressed *in situ* until the initial dislocation network structure formed. The sample was then transferred to a double-tilt holder for data collection. Diffraction-contrast electron tomography was used to generate 3D representations of the dislocation network structure, more details for reconstructing tomograms had been described previously^20^. Figure 2A–E shows the 3D structure of the dislocation net after reconstruction. Different views of the constructed 3D-dislocation model are shown along with the real space coordinate system. Figure 2F shows the loading schematic diagram of the nano-pillar. According to the tomogram, it was observed that the dislocations are all 〈a〉 types with Burgers vectors $\pm {\rm{a}}/3$[$2\bar{1}\bar{1}0$], $\pm {\rm{a}}/3$[$\bar{1}\bar{1}20$] and $\pm {\rm{a}}/3$[$\bar{1}2\bar{1}0$] (marked by lines in purple, green and yellow, respectively), which glide in the prismatic planes of titanium. The loading direction was almost perpendicular to a prismatic plane and was about 85 degrees off the [$\bar{1}2\bar{1}0$] direction (colored in yellow), indicating that the dislocations with $\vec{{\bf{b}}}$ = $\pm {\rm{a}}/3$[$\bar{1}2\bar{1}0$] have quite low mobility. In contrast, the other two types of dislocations can glide on their prismatic planes and the slip systems were identifies as ($01\bar{1}0$)[$2\bar{1}\bar{1}0$] and ($1\bar{1}00$)[$\bar{1}\bar{1}20$], respectively. The less mobile dislocations could pin the other two types of dislocations to hinder their motion, forming dislocation junctions. New dislocations were generated from the dislocation network, resulting in a rather dense dislocation structure ultimately.

It is worth mentioning that this orientation was chose to enable multiple slips in titanium. Apparently dislocation starvation was not observed. To test the impact of orientation on the ability of dislocation storage in small HCP titanium crystals, nano-pillars with the orientation that was about 10 degrees off the [0001] orientation were tested under which only one slip system was favored. The single-crystalline Ti nano-pillars were ~150 nm in diameter as well. The *in situ* compression tests were carried out under dark field STEM mode.

**Figure 3:**
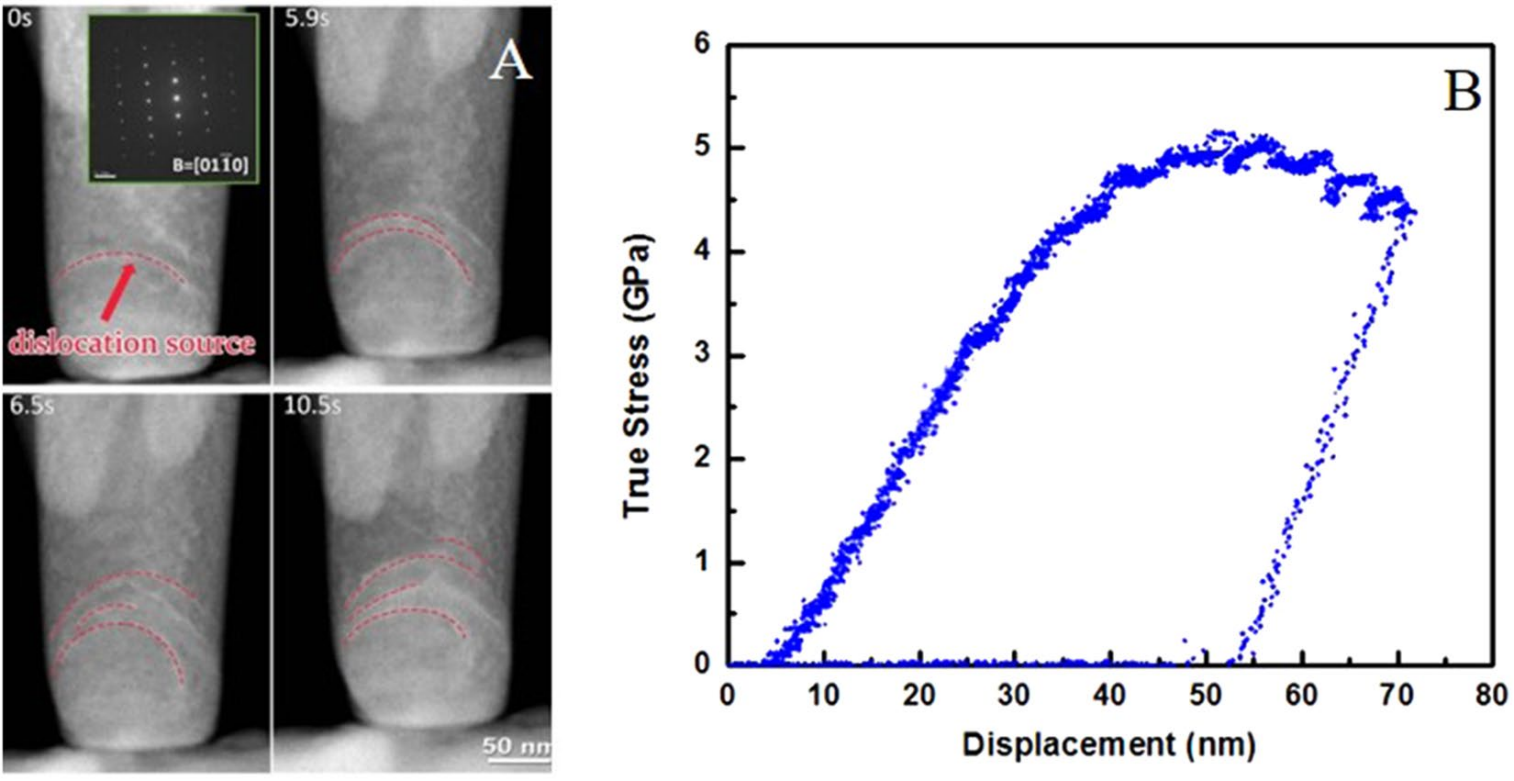
(**A**) TEM images from the movie of a micro-pillar compression test as the loading direction was 10 degrees off [$0001$] on a ~150nm-sized single-crystal Ti pillar, showing the process of dislocation multiplication with a dominant dislocation acted as a typical Frank-Read source. The zone axis was along [$01\bar{1}0$], the insect shows the electron diffraction pattern. (**B**) True Stress-Displacement curve of the quantitative *in situ* TEM nano-mechanical test. Continuous plastic flow and high strength was observed.

Figure 3A demonstrates an example of the deformation of the nanopillars with loading direction close to [$0001$] direction. In contrast, it was observed that there was a dominant dislocation source near the contact surface, which continuously generated the same type of dislocations (marked by the dash line in red). The new dislocations didn’t escape from the surface but formed dislocation array inside the pillar. No dislocation starvation phenomenon was observed as well. Figure 3B is the corresponding true stress-displacement curve showing the mechanical response. The curve shows a yield point at about 4 GPa, but no significant load drop was observed compared to Fig. 1B. After yielding, the stress increased with increasing strain, and the contact strength further increased to about 5 GPa. Interestingly the pillars loaded along both directions all yielded at the similar stress around 4 GPa and the maximum contact stress were all about 5 GPa, indicating the weak orientation dependence of the mechanical properties of the HCP titanium crystal at this extremely small scale.

To sum, the dislocation behaviors and the mechanical properties of the HCP structured titanium pillars are distinct from FCC and BCC materials at extremely small scale. Since dislocation interactions and multiplications still operate, dislocation starvation was not obvious and the strain bursts, which have been frequently observed in FCC and BCC materials at small scale, are not common in small HCP titanium crystals. Consequently the HCP titanium pillars show much better plastic stability at small scale and the structure rarely collapses.

Image force from the surfaces is one of the major driving forces for the escape of dislocations, which increases with decreasing sample size, while Peierls stress is the main resistance to dislocation motion. The physical mechanism for dislocation starvation can be understood in terms of a competition between the dislocation nucleation/activation rate and the (mobile) dislocation annihilation rate^18^. FCC metals have relatively high dislocation mobility because of the low Peierls stress and the existence of multi-slip systems. Since the critical stress needed for dislocation self-multiplication increases with decreasing size, dislocations escape from the crystal before multiplying and interacting with each other, resulting in dislocation starvation effect in small FCC pillars. In contrast, BCC metals have higher Peierls stress and easier cross-slip of screw dislocations compared to FCC metals in general^15^. However dislocation starvation is still a phenomenon in common in BCC metals at small scale. In some BCC metals such as Nb, the thermal fluctuation energy overwhelms the Peierls barrier at near room temperature; the materials exhibit FCC-like size effect and dislocation starvation. While in Mo, which has higher critical temperature, dislocation starvation becomes the dominant deformation mechanism as the size of samples reduce to very small (for example D < 200 nm) and the stress reaches sufficiently high to diminish the mobility difference between edge and screw dislocations^21^. Different from BCC and FCC metals, our experimental results indicate that HCP structured Ti can store dislocations even at extremely small scale.

In HCP structured metals, the Peierls stress for slip systems is usually even higher and the Burgers vectors of dislocations are relatively larger, indicating that the dislocation slip has lower mobility compared to that in cubic structured metals^22^. As the size of samples decrease to extremely small (around 200 nm in diameter and below), the yield stress increases to about 4–5 GPa and is almost orientation independent. The dislocation activities started with dislocation nucleation events under different loading directions, indicating that the critical stress for activating pre-existing dislocations already increases to the extremely high level of dislocation nucleation. The nucleation of dislocations with different Burgers vectors was observed, resulting in the formation of dislocation network that hindered dislocations from escaping from the surfaces. Even as the crystal orientation only favored single slip system, the dislocation mobility looked quite low and the dislocation lines moved extremely slowly once nucleated. As a result dislocation array formed in the small volume and the nucleation rate was obviously higher than the annihilation rate. Dislocation multiplication and interactions dominate the further plastic deformation, resulting in a significant increase of dislocation density. Without dislocation starvation, plastic flow was continues and relatively smooth even in the extremely small Ti pillars. The results demonstrated that HCP structured Ti has much better ability of dislocation storage and plastic stability at extremely small scale. It not only improved our understanding on the fundamental size effect on the dislocation behaviors and deformation of HCP structured metals but also shed light on the future applications of the related materials in nano-science and nano-technology.

## Methods

The crystal orientation of the Ti-5at%Al single crystal was determined using the Laue back reflection method. The pillar samples for compression testing were prepared using FIB. Compression testing was prepared using the Focused Ion Beam (FIB) micromachining technique. The samples have cylindric cross-sections with diameters of 150 nm. *In situ* nano-compression experiments were carried out at room temperature in a JEOL 3100 TEM operating at 300 kV and a Titan microscope at STEM mode with a Hysitron TEM PicoIndenter, respectively.

## Electronic supplementary material

Supplementary Information

movie1

movie2

movie3
