# Supplementary material for: Dislocation Multiplications in Extremely Small Hexagonal-structured Titanium Nanopillars Without Dislocation Starvation
Source: Sci Rep. 2017 Nov 21;7:15890. doi: 10.1038/s41598-017-16195-7 (PMC5698332; doi:10.1038/s41598-017-16195-7)
Supplement: Supplementary file 1 — Supplementary Information [file 41598_2017_16195_MOESM1_ESM.pdf]

SUPPLIMENTARY INFORMATION

Dislocation multiplications in extremely small hexagonal-structured titanium nanopillars without dislocation starvation

Peng Huang[1], Qian Yu[1,*]

[1]Center of Electron Microscopy and State Key Laboratory of Silicon Materials, Department of Materials Science and Engineering, Zhejiang University, Hangzhou 310027, China
[*]corresponding.kuw209@psu.edu, yu_qian@zju.edu.cn

Movies

1. Movie 1. In situ compression test of nano-pillar loaded 5 degrees off $[10\bar{1}0]$ direction. No dislocation was observed in the nano-pillar at the beginning. With further loading, several dislocation sources appeared near the contact surface which suddenly started to emit dislocations. The dislocations quickly intersected with each other and formed junctions, resulting in the formation of complex dislocation network ultimately.
2. Movie 2. 3D structure of the dislocation network. Different views of the constructed 3D-dislocation model are shown along with the real space coordinate system.
3. Movie 3. In situ compression test of nano-pillar loaded 10 degrees off [0001] direction. A dominant dislocation source near the contact surface was activated, which continuously generated the same type of dislocations. The new dislocations didn’t escape from the surface but formed dislocation array inside the pillar.
